# The tibialis posterior tendon footprint: an anatomical dissection study

**DOI:** 10.1186/s13047-020-00392-1

**Published:** 2020-05-19

**Authors:** Madeleine Willegger, Nargiz Seyidova, Reinhard Schuh, Reinhard Windhager, Lena Hirtler

**Affiliations:** 1 Department of Orthopedics and Trauma Surgery, Division of Orthopedics, Medical University of Vienna, Waehringer Guertel, 18-20 1090 Vienna, Austria; 2 Center for Anatomy and Cell Biology, Division of Anatomy, Medical University of Vienna, Vienna, Austria

**Keywords:** Tibialis posterior tendon, Anatomy, Footprint, Insertion, Flatfoot reconstruction, FDL transfer

## Abstract

**Background:**

The tibialis posterior tendon (TPT) is the main dynamic stabilizer of the medial longitudinal arch of the foot. Especially in adult acquired flatfoot deformity (AAFD) the TPT plays a detrimental role. The pathology and function of the tendon have been extensively investigated, but knowledge of its insertional anatomy is paramount for surgical procedures. This study aimed to analyze the complex distal footprint anatomy of the TPT.

**Methods:**

Forty-one human anatomical specimens were dissected and the distal TPT was followed to its bony footprints. After tendon removal the footprints were marked with ink. Standardized photographs were taken and consecutively analyzed by digital imaging measurements. Footprint length, width, area of insertion, location, and shape was studied regarding the main insertion at the navicular bone.

**Results:**

All specimens had the main TPT insertion at the navicular bone (41/41, 100%). Sixty-three percent of navicular TPT insertions were located at the plantar aspect. The mean navicular footprint measured 12.1 mm × 6.9 mm in length and width, respectively. The tendon further spread into several slips which anchored the tibialis posterior deep in the plantar arch. TPT insertions were highly variable with an involvement of up to eight distinct bony footprints in the mid- and hindfoot. The second most common additional footprint was the lateral cuneiform (93% of dissected feet), followed by the medial cuneiform (80%), the metatarsal bases [1–5] (80%), the cuboid (46%), the intermediate cuneiform (19%), and the calcaneus (12%).

**Conclusions:**

The present study adds to current knowledge on the footprint anatomy of the TPT. Based on the findings of this study we advocate a plantar location of flexor digitorum longus tendon transfer in flexible AAFD in order to restore the anatomical lever and insertion of the TPT.

## Background

The tibialis posterior tendon (TPT) elevates the medial arch and inverts, adducts, and plantar flexes the foot [1–3]. During the stance phase of gait the tibialis posterior is the main dynamic stabilizer of the foot. As a strong contributor to the midtarsal joint locking mechanism the TPT inverts the hindfoot, creating a rigid midfoot allowing the gastrocnemius-soleus complex to transmit plantar flexion forces to the metatarsal heads [4]. Posterior tibial tendon dysfunction (PTTD) is the prevailing cause of adult acquired flatfoot deformity (AAFD) which is characterized by a collapse of the medial longitudinal arch. Loss of tibialis posterior function enables hindfoot eversion, “unlocking” of the midtarsal joints and causing plantar flexion at the talonavicular joint as well as forefoot abduction [5]. TPT degeneration and elongation is associated with age-related AAFD, but multiple etiologies including traumatic TPT rupture have been identified [6–8]. Although controversy exists about the condition and its treatment, it is clear that the complex course and function of the TPT plays a detrimental role in the pathoanatomy of AAFD.

The anatomy of the TPT has been extensively investigated regarding its excursion, vascularity and tendon sheaths, but the insertional anatomy was disregarded by most of the studies so far [9–17]. The tibialis posterior muscle arises from the interosseous membrane and adjacent surfaces of the proximal tibia and fibula. The myotendinous junction appears in the distal third of the leg. The TPT courses behind the medial malleolus at a relatively acute angle and further passes posterior to the axis of the tibiotalar joint and medial to the axis of the subtalar joint [3, 4]. From an anatomical point of view, the main insertion of the TPT is at the navicular bone, but several additional insertions in the hindfoot and midfoot have been described [11, 14, 17]. Nevertheless, no study so far has reported the dimensional characteristics of the tendon footprint and the detailed bony insertional anatomy. More detailed knowledge about the normal anatomy of this tendon may aid to better understand its function and improve surgical reconstruction techniques by providing references for tendon reconstruction or tenodesis location.

The purpose of this study was to provide quantitative and qualitative information regarding the anatomical bony insertions of the tibialis posterior tendon and to quantify the prevalence of variations.

## Methods

Forty-one (41) adult formalin-fixed lower leg specimens were included in this study. The specimens were obtained from voluntary body donors who consented during life to donate their body for research and teaching purpose to the Center for Anatomy and Cell Biology, Division of Anatomy, Medical University of Vienna. The study has been approved by the local ethics committee (EK 1555/2015) prior to conducting the study. Specimen age ranged from 67 to 101 years (mean age 85.2 years). Twenty-six female and 15 male donor limbs were dissected including 20 left and 21 right lower legs. Inclusion criteria comprised specimens who were of sufficient quality without any evidence of surgical intervention in the area examined to allow for the complete identification of the TPT attachment. The skin, subcutaneous tissue and the muscles were removed with a scalpel. Care was taken to not injure the tibialis posterior muscle and its tendon. Each course of the TPT was documented by photograph according to a standardized protocol with a reference scale. A full-length photograph of the lower limb was taken from a medial view. Afterwards the foot was held in inversion in order to document the course of the TPT attaching at the medial and plantar aspect of the foot. Dissection was further continued and the main tendon and additional tendon slips were followed from proximal to distal exposing the bony attachments. The TPT was carefully dissected and then removed at the osseous insertions. The bony footprints were marked with ink and documented by photograph with a scale in a standardized manner [18, 19]. Photographs were taken from a plantar and medial view. The precise evaluation of the dimensions and shapes of the main navicular footprint was carried out after disarticulation of the bones followed by photography of the TPT insertion with a reference ruler. Qualitative analysis included the detection of variations and frequencies of bony TPT insertions in each disarticulated bone. Thereafter the photographs were digitally evaluated performing quantitative measurements of the footprint dimensions. The footprint length and width (in millimeters) were recorded, and areas of insertion (AOI, mm^2^) were calculated. All photographs were digitally measured by use of Image J (http://rsb.info.nih.gov/ij/) software. Image J is a Java-based image processing program developed at the National Institutes of Health (NIH) and is available for Microsoft Windows, the classic Mac OS, macOS, Linux, and the Sharp Zaurus PDA. The source code for Image J is freely available [20].

## Results

### Qualitative anatomy

**Fig. 1:**
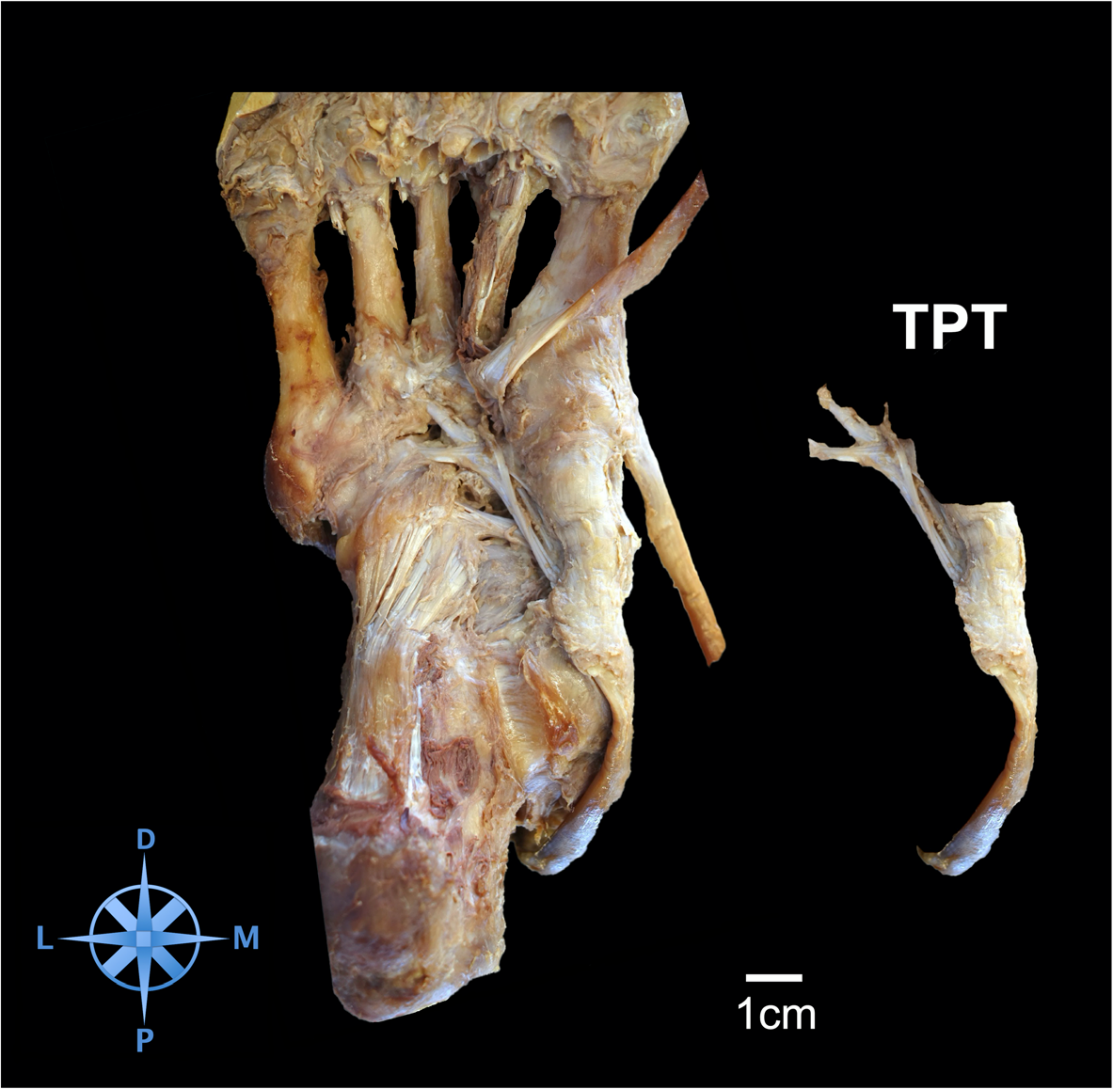
Plantar view of a right specimen before footprint dissection. The tibialis posterior tendon (TPT) is identified in the groove between the medial malleolus and the sustentaculum tali calcanei. The tendon can be followed to its insertion at the navicular bone. The tendon splits at the lateral plantar aspect of the navicular bone and spreads into several slips to additional bony insertion sites. On the right side the tendon with its slips is depicted. Scale: 1 cm; D = distal; M = medial; P = proximal; L = lateral

**Fig. 2:**
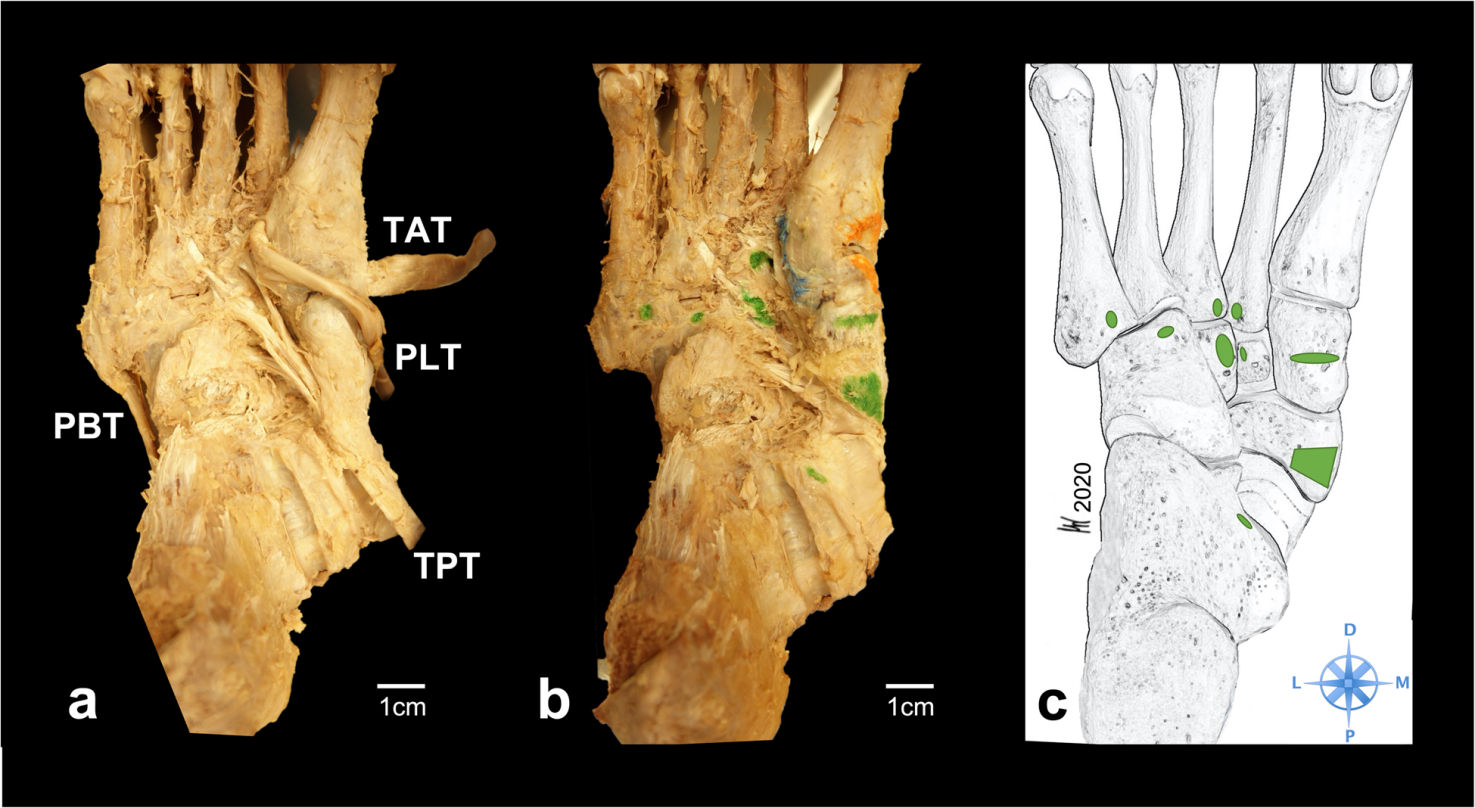
Exemplary specimen before and after footprint dissection. Plantar view of an exemplary specimen. **a**) skin, subcutaneous tissue and muscles have been removed in order to dissect the TPT. The peroneus longus tendon is retracted medially to visualize the plantar tendon slips of the TPT. PBT = peroneus brevis tendon, PLT = peroneus longus tendon, TAT = tibialis anterior tendon, TPT = tibialis posterior tendon **b**) the TPT footprints were marked with green ink. In this specimen the TPT inserted at the navicular bone, the medial, intermediate and lateral cuneiform, the cuboid, the calcaneus, and the 2nd, 3rd and 5th metatarsal. **c**) schematic drawing of the bony TPT footprints, D = distal; M = medial; P = proximal; L = lateral

**Fig. 3:**
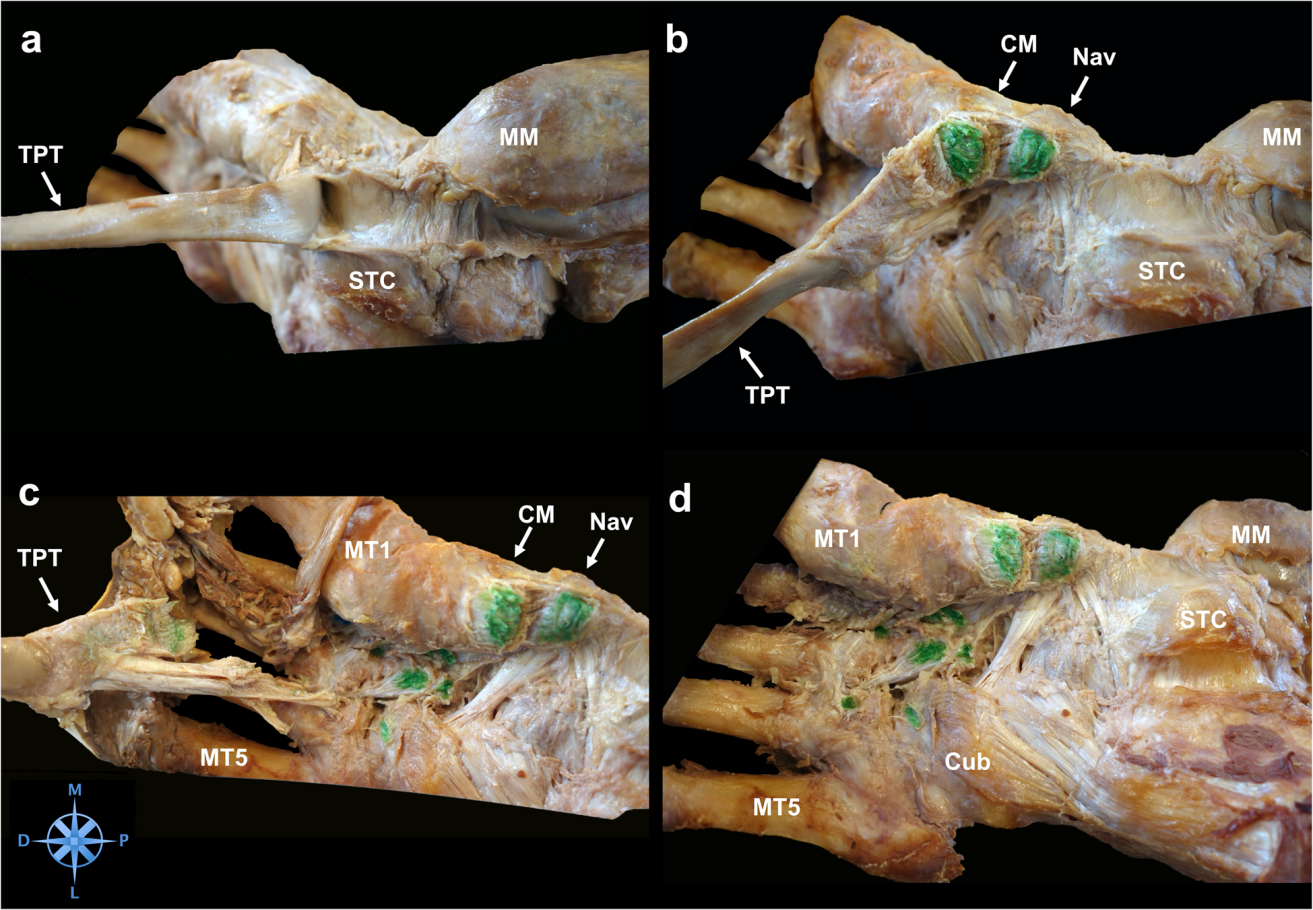
Step by step TPT footprint dissection. Figure 3**a** depicts the deflected and tensioned tibialis posterior tendon (TPT). Between the medial malleolus (MM) and the sustentaculum tali calcanei (STC) the tendon sheath of the TPT can be identified. **b** The first TPT insertion is located at the plantar aspect of the navicular bone (Nav). The tendon is cut from the bone and the footprint is marked with green ink. The next attachment of the TPT is found at the medial cuneiform bone (CM). **c** Another slip of the tendon dives deeper and laterally into the plantar tarsometatarsal region. The TPT is held under tension and all bony footprints are dissected and marked (green). MT1 = first metatarsal, MT5 = fifth metatarsal **d** All footprints at the plantar aspect of the foot are outlined: the navicular bone, the medial, intermediate and lateral cuneiform bone, the cuboid (Cub), the second and fourth metatarsal. M = medial; P = proximal; L = lateral; D = distal

**Fig. 4:**
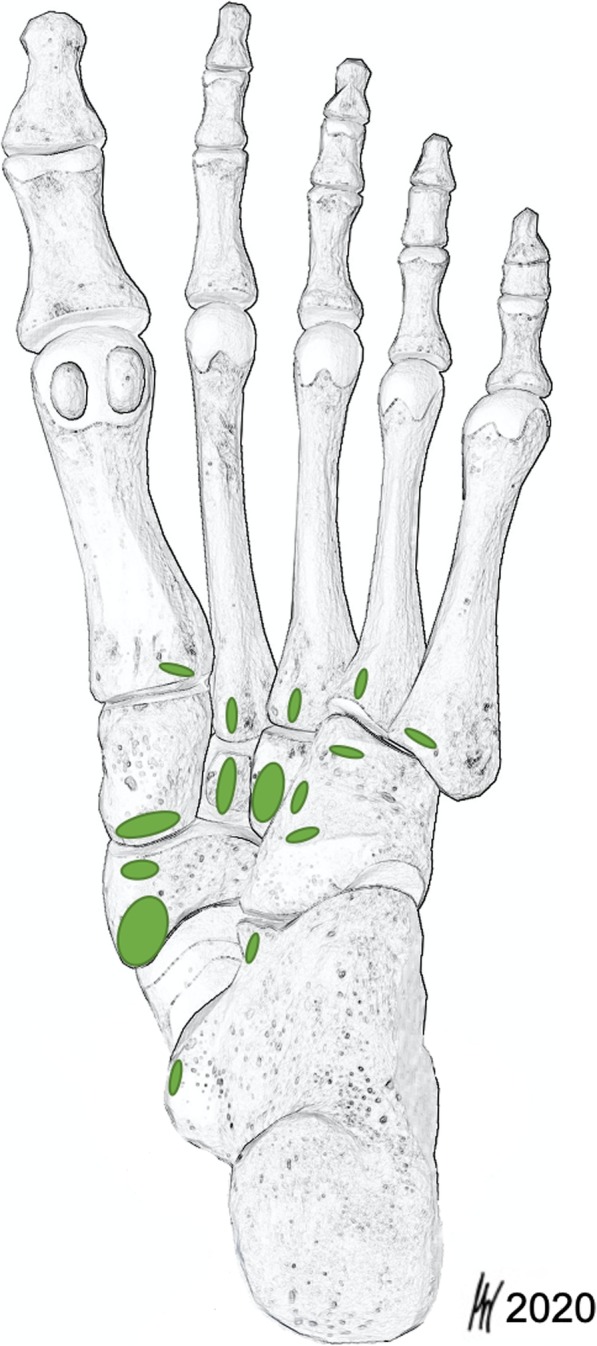
TPT footprints. Green ovals mark the possible TPT insertion sites. The navicular bone is the main insertion site but up to 8 distinct bony insertions of the TPT in a single foot have been detected. The second most common footprint was the lateral cuneiform (93%). At the cuboid the location of insertion was highly variable and at the calcaneus the anterior aspect of the sustentaculum tali and the distal medial distal aspect could be identified as PTT footprint location. All metatarsal bases can be involved in TPT anchoring

The TPT inserted in all specimens at the navicular bone (41/41, 100%). (Fig. 1) Distal to its insertion at the navicular bone the TPT spread further into several tendon slips and anchored the tendon deep in the longitudinal arch of the foot. (Fig. 2) In all dissected specimens we found between 2 and 8 distinct bony insertions involving the navicular bone, the medial cuneiform (80.5%), intermediate cuneiform (19.5%), and lateral cuneiform (92.7%), the cuboid (46.3%), the calcaneus (12.2%) and the bases of all metatarsals [1–5] (80.5%). (Figs. 3 and 4) Ainvolvement is outlined in Table 1.

**Table 1:** TPT footprint involvement of the metatarsal bases

Metatarsal	Count	Percent (%)
I	1	2%
II	12	21%
III	20	35%
IV	16	28%
V	8	14%
Total	57	100%

Table 1*: In 33 of 41 specimens (80.5%) footprints at the metatarsal bases were found. Various combinations of metatarsal bases involved in TPT insertion were detected. In total 57 metatarsal footprints in 41 feet were identified. The most common metatarsal footprint was located at the 3rd metatarsal base.*

**Fig. 5:**
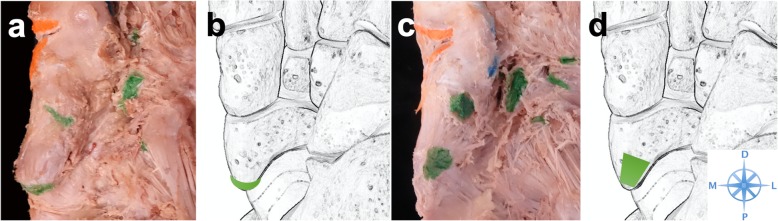
TPT footprint location at the navicular bone. This figure shows exemplary plantar views of dissected feet (**a**&**c**) accompanied by schematic drawings (**b**&**d**). The TPT footprints are marked with green ink. At the navicular bone the footprint was either located at the proximal apex of the tuberosity (a) (36.6%) or at the plantar (63.4%) aspect (c). D = distal; L = lateral; P = proximal; M = medial

At the main insertion at the navicular bone two locations could be identified. In 26 specimens (63.4%) we found a plantar location of the navicular footprint and in 15 (36.6%) feet the TPT inserted at the proximal apex of the tuberosity. (Fig. 5) In 2 specimens (4.9%) we found 2 separate footprints at the navicular bone.

**Fig. 6:**
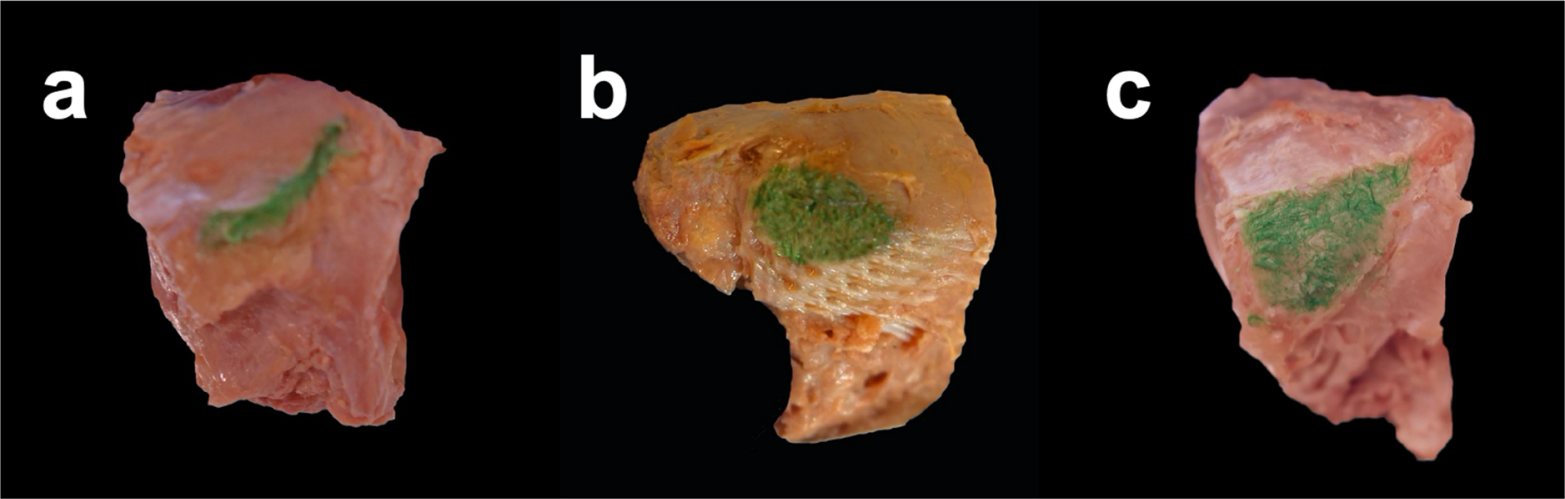
Footprint shape at the navicular bone. TPT footprints at the navicular bone are marked with green ink. Three different footprint shapes were identified: crescent (14.6%), oval (75.6%) and trapezoid (9.8%). **a**) right specimen with crescent shaped footprint, **b**) left specimen with oval shaped footprint, and **c**) right specimen with trapezoidal shaped footprint

The morphological shapes of the navicular TPT footprint were classified as oval, crescent, or trapezoid. The most common shape at the navicular bone was the oval type in 31 feet (75.6%) followed by the crescent type (6/41; 14.6%) and the trapezoid type (4/41; 9.8%), respectively. (Fig. 6).

### Quantitative anatomy

The mean length of the main bony insertion at the navicular bone was 12.1 mm (SD 3.3; range 6.1–21.8) and the mean width was 6.9 mm (SD 2.5; range 3.7–14.4), respectively. The mean area of insertion (AOI) was 72.8 mm^2^ (SD 33.6, range 23.1 - 142.7).

## Discussion

The most important finding of this study is that 63% of navicular TPT insertions were located at the plantar aspect. As the tendon dived deeper to the plantar side of the foot it spread into several branches which inserted at up to 8 distinct tarsometatarsal footprints. The second most common additional footprint was the plantar lateral cuneiform (93% of dissected feet). However, due to the complex course of the tendon slips and its high variability among the dissected specimens, we did not find it reasonable to classify different types of TPT insertions [11, 17]. Sarrafian described an anterior, middle, and posterior component of the TPT [17]. Other studies described a variable insertion pattern and supplementary attachments to the flexor hallucis brevis muscle, abductor hallucis muscle, peroneus longus tendon and to the spring ligament [11, 21]. Our study focused on the osseous footprints since these attachments represent the main anchorage of the tibialis posterior muscle.

From a biomechanical point of view, the course of TPT lies posterior to the axis of the tibiotalar joint and medial to the axis of the subtalar joint, allowing the muscle to act as a plantar flexor and invertor of the foot. During normal gait, the tibialis posterior acts to invert the hindfoot, causing the midtarsal joints to lock. In AAFD the valgus deformity of the hindfoot results from a collapse of the medial supporting TPT leading to an increased eversion of the calcaneus due to the position of the Achilles tendon lateral to the axis of the subtalar joint [2, 22]. According to this theory and applying tendon transfer principles, it has been postulated that the flexor digitorum longus (FDL) tendon, should be placed as far as possible from the subtalar joint axis (at the medial aspect of the navicular bone) in order to maximize leverage in FDL tendon transfer [4]. Nevertheless, following the anatomical course of the TPT, the first fulcrum of the TPT is the medial malleolar groove and the second fulcrum is the navicular tuberosity. Our study showed that the TPT primary inserts at the plantar aspect of the navicular bone and further spreads deeply, distally and laterally to additional bony insertions. With this anatomical knowledge we claim that the navicular tuberosity acts as an additional pivot point of the TPT to ease inversion of the foot. The course of the TPT with its attachment at the plantar aspect of the navicular bone works as a buttress for the medial longitudinal arch and the complex insertion at the plantar aspect of the foot provides a firm grip [23]. In stage II PTTD the flexor digitorum longus transfer replaces or supplements a pathologic TPT with the FDL. As both tendons are directly adjacent to each other posterior to the medial malleolus, they have the same line of pull. Based on the findings of this anatomical footprint study we advocate a plantar location of FDL tendon transfer in flexible AAFD in order to restore the anatomical lever and insertion of the TPT. If the FDL tendon is transferred too medially the direction of pull of the tendon alters and makes the FDL more an adductor of the foot rather than a supinator [24].

This principle is also applicable for suture anchor placement in the Modified-Kidner procedure. If the surgeon pursues an anatomical reconstruction, the anchor should be placed from plantar and perpendicular to the effective lever arm of the TPT from plantar medial to dorsal lateral [25].

This study comprises some inherent limitations. A comparison of left and right specimens was omitted due to the unpaired specimen study design. The described anatomical insertion of the TPT may vary according to the geographical origin, ethnicity, and the number of examined specimens. Nevertheless, the sample size of 41dissected feet constitutes a good sample size. Another potential drawback might be that the occurrence of an os tibiale externum was not analyzed. With a prevalence of 10 to 14% in normal feet the os tibiale externum is one of the most common accessory bones of the foot [26]. Therefore a huge amount of specimens would be necessary to analyze differences of TPT insertion in feet with normal navicular bones compared to type I-III accessory navicular bones. Additionally, pathological foot alignment (i.e. cavus or flatfoot) could be associated with anatomical variations of the TPT insertion. Evaluation of foot deformities was not part of the study protocol.

## Conclusions

In conclusion, this study adds to current knowledge on the anatomical insertion of the tibialis posterior tendon. The main footprint was located plantar at the navicular bone and several tendon slips extended up to 8 bony footprints located at the hind- and midfoot. The present data can be used as reference for anatomical TPT reconstructions or subsequently assist in surgical preparation of flexor digitorum longus transfer.

## Data Availability

The datasets generated and/or analyzed during the current study are not publicly available due to protection of privacy of body donors, but are available from the corresponding author on reasonable request.

## Abbreviations

AAFD: Adult acquired flatfoot deformity
FDL: Flexor digitorum longus
PTTD: Posterior tibial tendon dysfunction
TPT: Tibialis posterior tendon
